# Image Localized Style Transfer to Design Clothes Based on CNN and Interactive Segmentation

**DOI:** 10.1155/2020/8894309

**Published:** 2020-12-28

**Authors:** Hanying Wang, Haitao Xiong, Yuanyuan Cai

**Affiliations:** ^1^School of E-Business and Logistics, Beijing Technology and Business University, Beijing 100048, China; ^2^School of International Economics and Management, Beijing Technology and Business University, Beijing 100048, China

## Abstract

In recent years, image style transfer has been greatly improved by using deep learning technology. However, when directly applied to clothing style transfer, the current methods cannot allow the users to self-control the local transfer position of an image, such as separating specific T-shirt or trousers from a figure, and cannot achieve the perfect preservation of clothing shape. Therefore, this paper proposes an interactive image localized style transfer method especially for clothes. We introduce additional image called outline image, which is extracted from content image by interactive algorithm. The interaction consists simply of dragging a rectangle around the desired clothing. Then, we introduce an outline loss function based on distance transform of the outline image, which can achieve the perfect preservation of clothing shape. In order to smooth and denoise the boundary region, total variation regularization is employed. The proposed method constrains that the new style is generated only in the desired clothing part rather than the whole image including background. Therefore, in our new generated images, the original clothing shape can be reserved perfectly. Experiment results show impressive generated clothing images and demonstrate that this is a good approach to design clothes.

## 1. Introduction

In recent years, convolutional neural network has successfully completed a series of computer vision tasks, such as object detection, object recognition, image segmentation, and texture synthesis. In 2012, Krizhevsky et al. trained a large deep convolutional neural network and significantly improved the object recognition capability in the ImageNet challenge [1]. This has promoted many research studies and developments in the field of fashion, such as clothing classification and retrieval, clothing parsing, and recommendation. Yamaguchi et al. demonstrated an effective method for parsing clothing in fashion photographs and provided a large novel dataset and tools for labelling garment items to enable future research on clothing estimation [2]. Kalantidis et al. presented a scalable approach to automatically suggest relevant clothing products [3]. Liu et al. proposed a new deep model, namely, FashionNet, which learns clothing features by jointly predicting clothing attributes and landmarks [4]. The FashionNet develops powerful algorithms in clothes recognition and facilitates future research. Ma et al. proposed a novel fashion-oriented multimodal deep learning based model, bimodal correlative deep autoencoder (BCDA), to capture the internal correlation in clothing collocations [5].

In this paper, we focus on another purpose: the self-control of clothing style in local position. According to the trend of fashion, users select a clothing image as content image from Internet or shopping mall, and then find an art or a picture that they appreciate as a style image. Then, this algorithm is adopted to generate a unique clothing design, which combines the style of the style image and the clothing shape of content image. The term content refers to the shape of clothes, and the term style refers to patterns or colour in an image. In order to achieve the perfect preservation of clothing shape, we introduce a third image called outline image, which is extracted from content image by interactive GrabCut algorithm. The interaction consists simply of dragging a rectangle around the desired clothing. Then, we introduce an outline loss function based on distance transform of the outline image. In order to smooth and denoise the boundary region, total variation is employed. The proposed method allows users to interactively control the location of image style transfer and constrains that the new style is generated only in the desired clothing part rather than the whole image including background, which not only retains the basic shape of the original clothing but also designs a new style for it.

The main contributions are as follows. (1) People often encounter the situation that they like the shape of clothes but do not like the pattern or colour on the clothes. This research can customize styles for the same clothes to meet the fashion needs of customers. (2) The embarrassing scene of wearing the same dress is always annoying, but the expensive high-level customization is not available to every ordinary person. This research allows ordinary users who are not professional designers to easily design their own clothes, with low cost and high satisfaction of pursuing uniqueness. (3) This research can provide a lot of inspiration for professional designers. With the style given by style image, the design can be completed quickly, which improves efficiency and customer satisfaction.

## 2. Related Work

In the field of computer vision based on statistics, many scholars have begun to study image style transfer. Image style transfer can be considered as a process of image rendering, which is generally regarded as an extension of texture synthesis. Previous texture modelling methods mainly focus on parametric texture modelling with summary statistics [6–8] and nonparametric texture modelling with MRFs [9, 10]. The former one is to capture image statistics from a sample texture and exploit summary statistical property to model the texture [11]. The latter one is to use nonparametric resampling. A variety of nonparametric methods are based on the MRF model, which assumes that in a texture image, each pixel is entirely characterized by its spatial neighbourhood [11].

In 2015, Gatys et al. [12] proposed the first neural algorithm of artistic style, which can separate and recombine the content and style of natural images. They designed a model based on convolutional neural network, using pretrained VGG network to extract and store the features of images. Then, the total loss, which is a linear combination between the content and the style loss, is updated by backpropagation until the new image simultaneously matches the style features of the style image and the content features of the content image. Johnson et al. introduced perceptual loss functions for training feed-forward networks for image transfer tasks and greatly improved transfer speed [13]. Li et al. proposed a novel interpretation of neural style transfer by treating it as a domain adaptation problem and argued that the essence of neural style transfer is to match the feature distributions between the style images and the generated images [14]. However, these algorithms have some problems, such as the complexity of manual modulation and the instability of Gram matrix during optimization. Therefore, Chen et al. proposed StyleBank, which is composed of multiple convolution filter banks and each filter bank explicitly represents one style, for neural image style transfer [15]. This method, which runs in real time, is easy to train and produces results that are qualitatively better. Zhang and Dana found it fundamentally difficult to finish comprehensive style modelling using 1-dimensional style embedding. Therefore, they introduced CoMatch Layer that learns to match the second-order feature statistics with the target styles and built a multistyle generative network (MSG-Net), which achieves real-time performance [16].

The above algorithms achieve impressive results in style transfer, but with limited fidelity in local details, lacking good retention of content details in the process of style transfer and lacking semantic and depth information contained in content images. If applied to the fashion style transfer for clothing, the resolution of the generated clothing image will be very low, the shape of the clothing will be deformed, and the colour of the original clothing will be retained, which is difficult to combine with the new style. In order to achieve high fidelity of details, some scholars adopted patch-based approaches to image style transfer. Chen and Schmidt proposed a simpler optimization objective based on local matching that combines the content structure and style textures in a single layer of the pretrained network and then conducted the style swap between the content and the style [17]. Li and Wand introduced Markovian generative adversarial networks (MGANs) to capture the statistics of local patches and assemble them to high-resolution images [18]. Then, they combined generative Markov random field (MRF) models with discriminatively trained deep convolutional neural networks (dCNNs), which can both match and adapt local features with considerable variability [19].

Recently, deep learning methods have shown superior performance in analysis images [20–22]. Specially, Jiang and Fu introduced GAN to style transfer to address the challenge [23]. However, patch-based approaches well preserve both global and local structure only when the style and content images are with the similar structure such as face-to-face. It is difficult for style image to have the similar structure as the clothing image. They proposed an end-to-end feed-forward neural network which includes a fashion style generator and a discriminator. They combined global and patch-based methods, and the inputs included a set of clothing patches and full images. The clothing shape and design are preserved by the global optimization stage, and the detailed style pattern is preserved by the local optimization stage. Experiments and analysis show that their generated images are impressive and outperform other algorithms. However, their work still has some limitations. If a large area of clothing is plain and nontexture or does not have any patterns, the style texture on the clothing may fail to be generated. Furthermore, the generation of method based on GAN is not stable. The new image has high resolution only when spanning space is well constrained. In additional, generative adversarial network, as a data-driven method, has to obtain plenty of data. When applied to fashion style transfer, it is very inconvenient for ordinary users to design their own new clothes.

Different from other research studies, this paper applies the neural style transfer to fashion field, enabling ordinary users to design their own unique clothing according to their preferences. The input of this approach can be a clothing image with complex background rather than a simple clothing image without person and background. The paper focuses on users controlling the local transfer position of an image, facilitating the operation and greatly making it convenient for general public to design a clothing style. The research aims to achieve the perfect preservation of clothing shape, obtaining a new clothing design that simultaneously matches the style of the style image and the clothing shape of the content image.

## 3. Image Style Transfer

**Figure 1:**
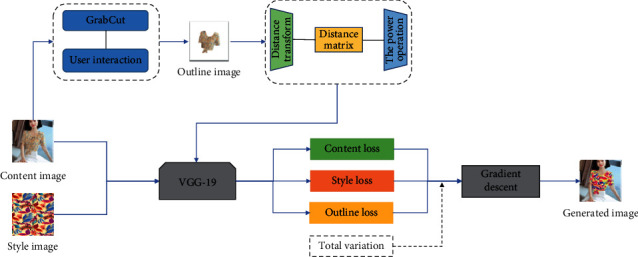
The framework of our proposed style transfer algorithm.

Figure 1 shows the process of image style transfer in this paper. (1) Using pretrained VGG network to extract and store the features of content and style images. (2) Obtaining an outline image by using GrabCut algorithm to segment content image, by simply dragging a rectangle around the desired clothing. (3) Obtaining the distance transform matrix by the distance transform of the outline image. (4) Obtaining outline feature by taking pixelwise power of distance transform matrix to emphasize the specific group of pixels. (5) Calculating content, style, and outline loss based on their features. (6) Adding total variation to total loss, which is a linear combination between the content, style, and outline loss.

### 3.1. Image Segmentation

An image is divided or partitioned into various parts called segments based on its colour, grey, texture, and other features so that these features show differences between different areas but show similarities in the same areas. At present, the main image segmentation algorithms include segmentation based on region, which divides the objects into different parts based on similarity of grey distribution, segmentation based on threshold, which divides the objects into different parts based on grayscale of pixels, segmentation based on edge which uses an image's discontinuous local features to detect edges and thus define a boundary of the object, and segmentation based on clustering which separates the pixels of the image into homogeneous clusters.

The above algorithms all have been widely used for image segmentation in different fields. They are simple but powerful methods for separate objects from background. However, when there are several similar targets in the same background, such as one person with T-shirt and trousers, they will separate the whole figure from background rather than only T-shirt or only trousers. Therefore, in order to make users specifically control the local transfer position of an image and to make the operation as simple as possible, this paper adopts interactive GrabCut algorithm [24] in the handling of segmentation. GrabCut algorithm is an improvement on GraphCut algorithm [25]. GraphCut algorithm needs to mark both “object” and “background” segments. Hard constraints for segmentation are imposed by indicating certain pixels that absolutely have to be part of the object and certain pixels that have to be part of the background [25]. But GrabCut algorithm needs significantly fewer interactions. The interaction consists simply of dragging a rectangle around the specific clothing that requires style transfer. Pixels outside the rectangle are marked as known background, and pixels inside are marked as unknown, which imposes certain hard constraints on segmentation. Then, a model is created to determine whether the unknown pixels are foreground or background.

#### 3.1.1. The GrabCut Segmentation Algorithm

The image is regarded as an array *Z* = (*z*_1_,…, *z*_*n*_,…, *z*_*N*_) of grey values in graph cut algorithm, and this array is used to describe an image in the color space. The values of the pixels are expressed as an array of “opacity” value *α* = (*α*_1_,…, *α*_*n*_), *α* ∈ [0, 1], with 0 being for background and 1 being for foreground. In the GrabCut algorithm, the image is taken to consist of pixels *Z*_*n*_ in RGB colour space through creating multivariate GMMs (Gaussian mixture models) with *K* components for background and foreground regions. A vector *k* = {*k*_1_,…, *k*_*n*_,…, *k*_*N*_) is introduced, with *k*_*n*_ ∈ {1,…, *K*} assigned to each pixel.

The Gibbs energy of GrabCut algorithm for segmentation becomes

(1) $$\begin{array}{c} E\left(\alpha ,k,\theta ,z\right)=U\left(\alpha ,k,\theta ,z\right)+V\left(\alpha ,z\right), \end{array}$$

where *E* is defined as Gibbs energy, *U* is the data term to evaluate the fit of the opacity distribution *α* to the data *z*, *V* is the smoothness term, and the parameter *θ* describes grey-level distributions of image foreground and background and consists of histograms of grey values as equations (2)–(4):

(2) $$\begin{array}{c} \theta =\left\{h\left(z;\alpha \right),\;\alpha =\;0,1\right\}, \end{array}$$

(3) $$\begin{array}{c} U\left(\alpha ,k,\theta ,z\right)=\sum_{n}D\left(\alpha_{n},k_{n},\theta ,z_{n}\right), \end{array}$$

where

(4) $$\begin{array}{c} D\left(\alpha_{n},k_{n},\theta ,z_{n}\right)=-\text{log}p\left(z_{n}|\alpha_{n},k_{n},\theta \right)-\text{log}\pi \left(\alpha_{n},k_{n}\right), \end{array}$$

where *p*(·) is a Gaussian probability distribution and *π*(·) is the mixture weighting coefficient. So,

(5) $$\begin{array}{c} D\left(\alpha_{n},k_{n},\theta ,z_{n}\right)=-\text{log}\pi \alpha_{n},k_{n}+\;\frac{1}{2}\text{log det}\Sigma \alpha_{n},k_{n}+\frac{1}{2}{\left[z_{n}-µ\alpha_{n},k_{n}\right]}^{T}\sum \alpha_{n},k_{n}^{-1}\;\left[z_{n}-µ\alpha_{n},k_{n}\right]. \end{array}$$

Therefore, the parameters of the model are as follows:

(6) $$\begin{array}{c} \theta =\left\{\pi \left(a,k\right),\mu \left(a,k\right),\Sigma \left(a,k\right),\alpha =0,1,k=1,\ldots ,k\right\}, \end{array}$$

where the parameter *θ* depends on the weights *π*, means *μ*, and covariances Σ After learning the three parameters, we can get the grey-level distributions of image foreground and background. The smoothness term *V* is computed by using Euclidean distance as follows:

(7) $$\begin{array}{c} V=\left(a,z\right)Y\sum_{m,n\in C\;}\left[\alpha_{n}\neq \alpha_{m}\right]\text{exp}-\beta {\left\|z_{m}-z_{n}\right\|}^{2}, \end{array}$$

where *C* is the set of pairs of neighbouring pixels. This value of constant *β* ensures that the exponential term switches appropriately between high and low contrast.

The Gibbs energy function helps to determine whether the unknown pixels are foreground or background and also shows the difference between neighbouring pixels of an image. The algorithm finally gets the local minimum which converges at least to *E*. It can straightforwardly detect when *E* stops to decrease significantly and to terminate iteration automatically [24].

#### 3.1.2. Results of GrabCut

**Figure 2:**
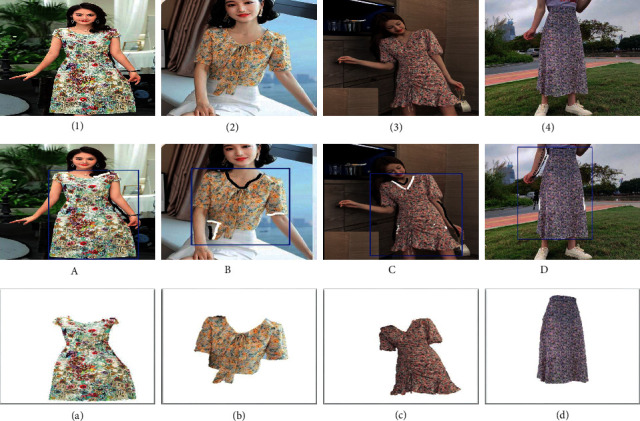
Outline images in the bottom row are extracted from content images in the top row by interactive GrabCut algorithm. Images in the middle row display all user interactions: black (background brush); white (foreground brush).

Users can employ GrabCut algorithm, an interactive image segmentation method, to specifically extract any part of an image, which is in line with our purpose of extracting desired clothing from complex background to generate outline image. The results are shown in Figure 2.

Through our experiments, we find that in these four cases, the bounding rectangle alone is not sufficient to enable foreground extraction to be completed. The models' necks or arms are dragged into the rectangle, so sometimes they are mistakenly regarded as foreground. Therefore, we have to make some simple brush strokes on the clothing parts. Black means selecting areas of background, and white means selecting areas of foreground. According to Figure 2, we can see that there is no need for complicated strokes. Users simply brush the boundaries with a few strokes. Therefore, the operation is acceptable.

### 3.2. Style Transfer

The basic principle of neural style transfer is to extract the content features and style features of the input images and to use the pretrained convolutional neural network (CNN) to mix them to generate new images. In this paper, input images include content image with complex background, outline image that is extracted from content image by GrabCut, and style image. They are input into the convolutional neural network, and by constraining the content loss, style loss, and outline loss, a stylized clothing image *G* is generated, which simultaneously matches the shape of the original clothing and the style of the style image. In this paper, the trained VGG-19 network model is used as the image feature extractor [26].

#### 3.2.1. Content and Style Loss

With a given input image to the CNN, filter responses to every layer are produced as feature map. Feature maps on some layers can be regarded as the content representation. Gatys et al. considered the feature responses in higher layers of the network as the content representation [12]. The layer *l* with *N*_*l*_ distinct filters has *N*_*l*_ feature maps, and *M*_*l*_ describes the height times the width of the feature map. Therefore, the content representation of an image in the layer *l* can be defined as a matrix *F*^*l*^ ∈ *R*^*N*_*l*_×*M*_*l*_^, where *F*_*ij*_^*l*^ is the activation of the *i*^th^ filter at position *j* in layer *l*. Therefore, content image $\overset{⟶}{p}$ and the random noise image $\overset{⟶}{x}$, which is obtained by initializing content image through network, have their respective content feature representation *P*^*l*^ and *F*^*l*^ in layer *l*. Content loss between the two feature representations is defined as follows:

(8) $$\begin{array}{c} L_{c}\left(\overset{⟶}{p},\overset{⟶}{x},1\right)=\frac{1}{2}\sum_{i,j}{\left(F_{ij}^{l}-p_{ij}^{l}\right)}^{2}. \end{array}$$

The derivative of this loss is defined as follows:

(9) $$\begin{array}{c} \frac{\partial L_{c}}{\partial F_{ij}^{l}}=\left\{\begin{array}{cc} {\left(F^{l}-P^{l}\right)}_{ij}, & \text{if }F_{ij}^{l}>0,\\ 0, & \text{if }F_{ij}^{l}<0. \end{array}\right. \end{array}$$

To obtain style representation of an input image, Gatys et al. used a feature space. This feature space is built on top of the filter responses in any layer of the network to capture texture information. According to Gatys, textures of an image are per definition stationary, so a texture model needs to be agnostic to spatial information [12]. Thus, our style representation should discard the spatial information in the feature maps, which is given by the correlations between the different filter responses [12]. These feature correlations are obtained by the Gram matrix *G*^*l*^ ∈ *R*^*N*_*l*_×*M*_*l*_^, where *G*_*ij*_^*l*^ is the inner product between the feature maps *i* and *j* in the layer *l* as shown in the following equation:

(10) $$\begin{array}{c} G_{ij}^{l}=\sum_{k}F_{ik}^{l}F_{jk}^{l}, \end{array}$$

where *k* represents the *k*^th^ element of the feature map. A set of Gram matrices {*G*^1^, *G*^2^,…, *G*^*l*^} from layers 1, 2,…, *l* in the network in response to a given style image provides a stationary description of the texture, which fully specifies a style in our model. To generate a new style on the basis of a given image, we use a random noise image to find another image that matches the Gram-matrix representation of the original image by using gradient descent. And the optimization is done by minimizing the mean-squared distance between the entries of the Gram matrix of the generated image and the Gram matrix of the original image. Style image $\overset{⟶}{a}$ and the random noise image $\overset{⟶}{x}$ have their respective style feature representation *A*_*l*_ and *G*_*l*_ in layer *l*. The contribution of layer *l* to the total loss is expressed as follows:

(11) $$\begin{array}{c} E=\frac{1}{4N_{l}^{2}M_{l}^{2}}\sum_{i,j}{\left(G_{ij}^{l}-A_{ij}^{l}\right)}^{2}, \end{array}$$

and the total style loss is

(12) $$\begin{array}{c} L_{s}\left(\overset{⟶}{a},\overset{⟶}{x}\right)=\sum_{l=0}^{L}w_{l}E_{l}, \end{array}$$

where *w*_*l*_ are weighting factors of the contribution of each layer to the total loss. The derivative of *E*_*l*_ can be computed as follows:

(13) $$\begin{array}{c} \frac{\partial E_{l}}{\partial F_{ij}^{l}}=\left\{\begin{array}{cc} \frac{1}{N_{l}^{2}M_{l}^{2}}{\left({\left(F^{l}\right)}^{T}\left({\text{G}}^{l}-A^{l}\right)\right)}_{ji}, & \text{if }F_{ij}^{l}>0,\\ 0, & \text{if }F_{ij}^{l}<0. \end{array}\right. \end{array}$$

#### 3.2.2. Outline Loss

With neural style transfer, the style from the style image is transferred to the whole content image. However, in the fashion field, in order to get a desired clothing design, the following two points must be constrained. Firstly, keep the shape of the original clothes; secondly, ensure that the style is only transferred to the desired clothes in the content image, and the rest of the areas keep clean.

**Figure 3:**
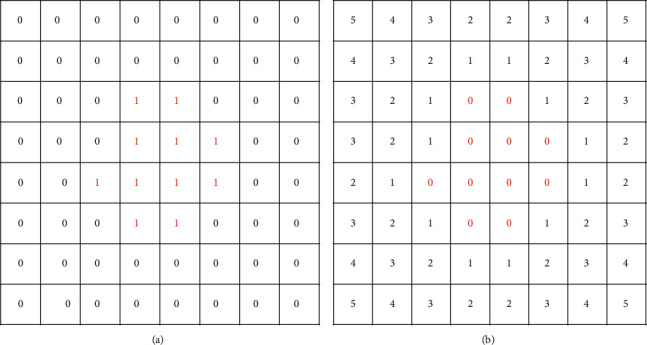
(a) The input outline image and (b) the distance transform result.

For decorated logo generation, Atarsaikhan et al. proposed a new loss function based on distance transform of the input image, which allows the preservation of the silhouettes of text and objects, constraining style transfer only around the designated area [27]. Therefore, based on this method, we introduce an outline loss function, which is obtained by converting outline image to binary image and then conducting distance transform. The concept of distance transform was first proposed by Rosenfeld and Pfaltz [28]. The basic idea is to convert a binary image into grayscale image where each object pixel has a value corresponding to the minimum distance from the background. Take binary image as an example (Figure 3), where the pixel values inside the outline are 1, and the other pixel values are 0. Distance transform calculates the distance between each pixel and the nearest outline boundary, and the pixel values inside the outline become 0.

In this paper, the outline image pixels are divided into the inside pixels *p* of the outline and the outside pixels *q* of the outline. By calculating the distance between the inside pixels and the outside pixels in Euclidean distance metric, the distance matrix *D* is obtained as follows:

(14) $$\begin{array}{c} Dp\;,q=\text{Min}\left(\text{disf}\left(p,q\right)\right), \end{array}$$

(15) $$\begin{array}{c} \text{disf}\left(p\left(x_{1},y_{1}\right),q\left(x_{2},y_{2}\right)\right)=\sqrt{{\left(x_{1}-x_{2}\right)}^{2}+{\left(y_{1}-y_{2}\right)}^{2}}. \end{array}$$

The farther the distance, the larger the value of the pixel, the clearer the outline, and the better the shape of clothes. With distance transform, the values of the inside pixels of outline become zero. Therefore, in order to increase the distance, we take pixelwise power of outside pixels of outline, with power of two or more [27] as follows:

(16) $$\begin{array}{c} d_{ij}=\left\{\begin{array}{cc} 0, & \text{if inside of the outline,}\\ d_{ij}^{n}, & \text{otherwise}. \end{array}\right. \end{array}$$

Through emphasizing the specific group of pixels, we get the distance feature. Given a random noise image $\overset{⟶}{x}$ and an outline image $\overset{⟶}{o}$, the distance feature is $D_{\overset{⟶}{x}c}$ and $D_{\overset{⟶}{o}c}$. The distance loss *L*_*d*_ is defined as follows:

(17) $$\begin{array}{c} L_{d}=\frac{1}{2}\;{\left(D_{\overset{⟶}{x}c}^{n}-D_{\overset{⟶}{o}c}^{n}\right)}^{2}. \end{array}$$

#### 3.2.3. Style Transfer

In this paper, style transfer is guided by the difference between the Gram-matrix representation of the content image, the style image, the outline image, and the generated image. The difference is represented by the loss function.

The loss functions used in this paper include content loss *L*_*c*_ for preserving the content of the content image, style loss *L*_*s*_ for preserving the style of the style image, and outline loss *L*_*d*_ for preserving the original shape of clothes and limiting the style transfer region. Suppose that *α*, *β*, and *γ* are the weighting factors for them, respectively; then, *L*_total_ is defined as follows:

(18) $$\begin{array}{c} L_{\text{total}}=\alpha L_{c}+\beta L_{s}+\gamma L_{d}. \end{array}$$

#### 3.2.4. Total Variation Regularization

In order to smooth and denoise the boundary region, total variation norm *R*_TV_ is employed. Then, we can combine total variation norm *R*_TV_ and the total loss functions into total loss *L*_total_ by taking linear addition. In this paper, KL diversity, which is commonly used in image classification and prediction, is chosen as the loss function in the proposed method.

(19) $$\begin{array}{c} R_{\text{TV}}=\frac{D_{x}^{2}}{N_{D_{x}}}+\frac{D_{y}^{2}}{N_{D_{y}}}, \end{array}$$

where *D*_*x*_ and *D*_*y*_, respectively, represent the transverse difference and longitudinal difference of the generated image, while *N*_*D*_*x*__ and *N*_*D*_*y*__ represent the number of elements of the corresponding difference results.

## 4. Experimental Results

### 4.1. Dataset and Experimental Set

Higher layers in the network capture the high-level content in terms of objects and their arrangement in the input image but do not constrain the exact pixel values of the reconstruction [12]. In contrast, reconstructions from the lower layers simply reproduce the exact pixel values of the original image [12]. Therefore, we use different layers to extract features. In this paper, the pretrained VGG-19 network is applied, consisting of 16 convolutional layers and 5 pooling layers. We reconstruct the content of the input images from layers conv4-2 and reconstruct the style of the input images from layers conv1-1, conv2-1, conv3-1, conv4-1, and conv5-1. Weighting factors for content image and style image, *α* and *β*, are 0.001and 0.8, respectively. *γ* is 1.0, and the weight of R_TV is 0.001.

**Figure 4:**
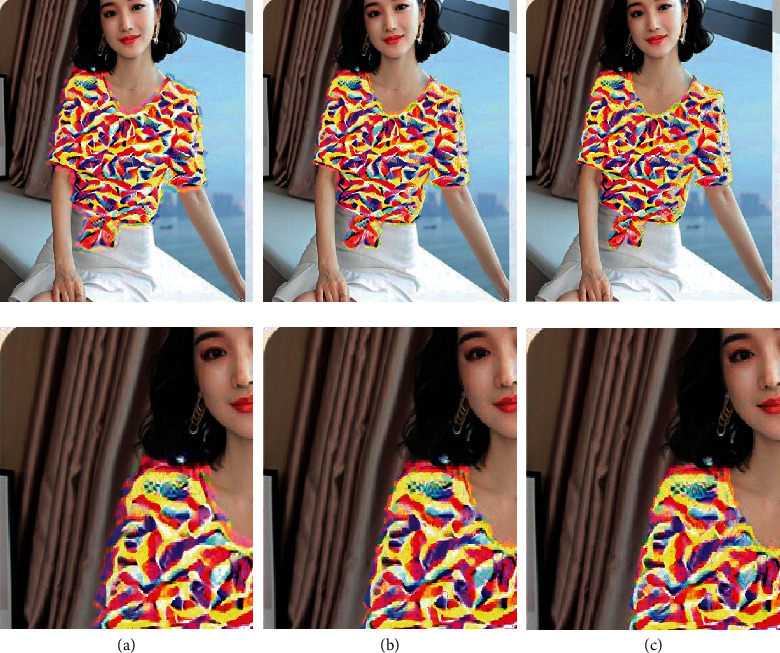
The results with different values of weighting factor *γ*. When increasing the values of *γ*, the noise is removed a lot. (a) *γ* = 0.00001. (b) *γ* = 0.01. (c) *γ* = 1.0.

Figure 4 shows different values of weighting factor *γ*. Outline loss *L*_*d*_ is designed to preserve the shape of clothing. Through experiments, we can see that with the increase of weighting factor *γ*, the noises from outside of the clothing shapes can be removed. Therefore, style transfer can be more tightly constrained within the outline area.

**Figure 5:**
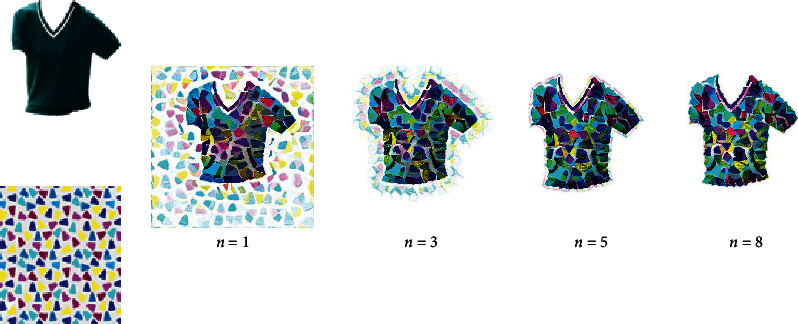
The results with different emphasizing power *n*. When there is no emphasis (*n* = 1), there is a lot of noise around the clothes. When increasing the emphasizing power *n*, the noise is reduced a lot.

Figure 5 shows results with different emphasizing power *n*. When there is no emphasis (*n* = 1), there is a lot of noise around the clothes. When increasing the emphasizing power *n*, the noise is reduced a lot. Therefore, we can assume that the larger the emphasis on power, the more impressive the generated clothes' design. By taking pixelwise power of a matrix, the values of the outside pixels of outline become larger, and the values of the inside pixels of outline keep zero, which greatly enlarges the difference and separates the clothes and background.

The experimental pictures selected in this paper are from pop-fashion.com and Taobao.com.

### 4.2. Comparison with Other Methods

**Figure 6:**
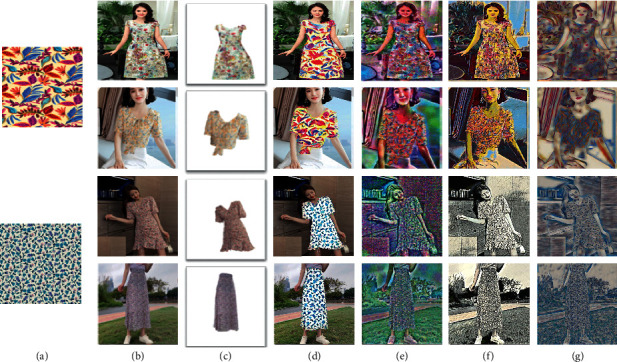
The first left column shows the input style images. The second left column shows four input content images. In our method, given style images, content images, and outline images of third column, new designs of clothes are generated. In other three methods, given style images and content images, new designs of clothes are generated. (a) Style image. (b) Content image. (c) Outline image. (d) Ours. (e) Gatys et al. [12]. (f) Zhang and Dana [16]. (g) Chen and Schmidt [17].

In Figure 6, we compare our method with other three types of style transfer methods. (1) NeuralST [12]: Gatys et al. showed artistic neural style transfer by generating a new image that combines both the content of the content image and the style of the style image. (2) MSG-Net [16]: Zhang and Dana built a multistyle generative network, which achieves real-time performance. (3) Style swap: Chen and Schmidt proposed a simpler optimization objective based on local matching that combines the content structure and style textures in a single layer of the pretrained network [17].

When comparing these methods, we find that only our method keeps the background clean. In other methods, the style from the style image is transferred to the whole content image rather than only clothes. Compared with Gatys, our method appears to better preserve the detailed textures of the style images and have less noise. The flowers and leaves of style images are very clear. Compared with Zhang and Dana, our method better transfers the colour of the style image. In the second last column, although the generated images preserve the textures of style images, they may lose the colour feature. In the last column, no matter the feature of content or style, they are both not well synthesized. However, in our method, the patterns of style images and the original global structure are faithfully blended.

### 4.3. Experimental Results

In order to show better results and make more sense, we have added more experiments and showed more examples. We picked several arts with different styles, like abstract arts, watercolour paintings, and oil paintings, and thus we got different effects.

**Figure 7:**
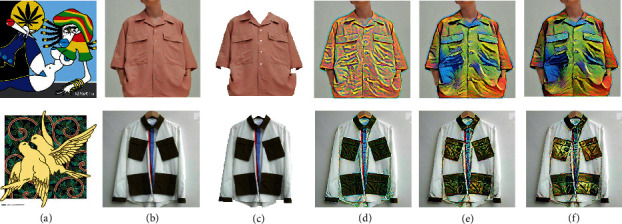
The style transfer results with images containing big and specific patterns as style images. (a) Style image. (b) Content image. (c) Outline image. (d) Iteration 100. (e) Iteration 600. (f) Iteration 1000.

Firstly, we picked two style images with big and specific patterns and picked two shirts as content images. We found it difficult to transfer such big patterns or figures from style images to clothes. However, the second line shows that the colour boundary can be identified automatically, so with different colour in original clothes, the style can be transferred variously. The results are shown in Figure 7.

**Figure 8:**
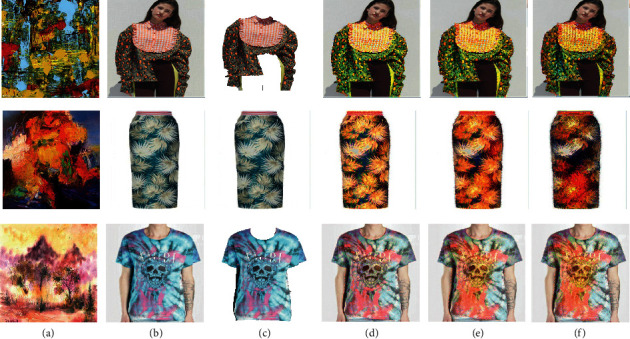
The results with oil paintings and watercolour paintings as style images. (a) Style image. (b) Content image. (c) Outline image. (d) Iteration 100. (e) Iteration 600. (f) Iteration 1000.

Secondly, in Figure 8, we picked two oil paintings and one watercolour painting as style images. These paintings do not have specific patterns, so these new images also do not generate any new ones. Fortunately, we can see that the style of painting, including colour, has been transferred to clothes perfectly and becomes a new and beautiful design.

**Figure 9:**
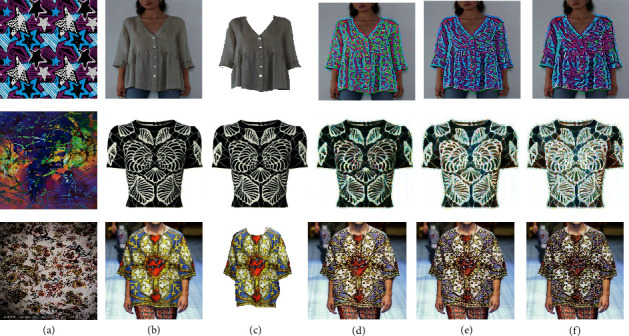
The results with random Internet pictures as style images. (a) Style image. (b) Content image. (c) Outline image. (d) Iteration 100. (e) Iteration 600. (f) Iteration 1000.

Thirdly, the three style images are random Internet photos. We can see that the colour has been transferred to clothes perfectly, and the new patterns will be randomly generated depending on the original ones in style images.  Figure 9 shows the results.

**Figure 10:**
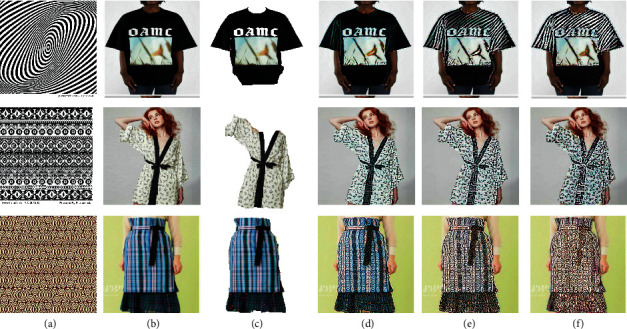
The results with images containing stripes as style images. (a) Style image. (b) Content image. (c) Outline image. (d) Iteration 100. (e) Iteration 600. (f) Iteration 1000.

Lastly, three style images with various stripes are used in Figure 10, and the results show that both the shape and the colour of stripes can be transferred perfectly to clothes. We conclude that the more specific and smaller the patterns are in style images, the more perfect they can be transferred.

## 5. Conclusion

This paper proposed an interactive image localized style transfer method especially for clothes. Through combining users' favourite clothing images and style images, a new design of clothes can be generated, which allowed ordinary users to easily design their own clothes and also provided inspiration for professional designers. In order to keep the shape of the original clothes and focus on localized transfer, we introduced a third image called outline image, which was extracted from content image by interactive GrabCut algorithm. Finally, the experiment results showed that the images generated by the method in this paper outperformed other methods. The approach in this paper has simple operations, high efficiency, and certain practicability.

Given the simplicity of our method, we believe that there is still substantial room for improvement. In future works, we plan to explore more advanced algorithm to allow more kinds of pictures, including both realistic and artistic images, as style inputs. More diverse image datasets will be used for training and testing to make the generated clothing styles more fashionable and more impressive.

## Data Availability

All data included in this study are available from the corresponding author upon request.
